# Potential impact of omentin-1 genetic variants with the clinical features and progression of buccal mucosa cancer

**DOI:** 10.7150/ijms.117708

**Published:** 2025-09-03

**Authors:** Ya-Hsin Wu, Chiao-Wen Lin, Hsiao-Chi Tsai, Chun-Wen Su, Ming-Yu Lien, Shun-Fa Yang, Chih-Hsin Tang

**Affiliations:** 1 School of Dentistry, China Medical University, Taichung, Taiwan.; 2 Department of Periodontology, China Medical University Hospital, Taichung, Taiwan.; 3 Institute of Oral Sciences, Chung Shan Medical University, Taichung, Taiwan.; 4 Department of Dentistry, Chung Shan Medical University Hospital, Taichung, Taiwan.; 5 Department of Medicine Research, China Medical University Beigang Hospital, Yunlin, Taiwan.; 6 Institute of Medicine, Chung Shan Medical University, Taichung, Taiwan.; 7 Department of Medical Research, Chung Shan Medical University Hospital, Taichung, Taiwan.; 8 School of Medicine, China Medical University, Taichung, Taiwan.; 9 Division of Hematology and Oncology, Department of Internal Medicine, China Medical University Hospital, Taichung, Taiwan.; 10 Department of Pharmacology, School of Medicine, China Medical University, Taichung, Taiwan.; 11 Department of Medical Laboratory Science and Biotechnology, College of Medical and Health Science, Asia University, Taichung, Taiwan.; 12 Chinese Medicine Research Center, China Medical University, Taichung, Taiwan.

**Keywords:** Omentin-1, genetic polymorphisms, oral squamous cell carcinoma

## Abstract

Oral cancer is the fourth most prevalent cancer among Taiwanese men and the ninth most general cancer among men globally. Omentin-1, an adipokine, has been shown to play a protective role by reducing proinflammatory cytokine secretion. The relationships between carcinogenic lifestyle factors, *OMNT1* polymorphisms, and oral squamous cell carcinoma (OSCC) remain unclear. We investigated the impact of clinicopathological features and four *OMNT1* gene variants on oral cancer risk in 406 Taiwanese male patients with the condition. Compared with the TT genotype, the TA+AA genotypes of SNP rs2274907 were linked with an increased risk of advanced clinical stage (III+IV). In patients with OSCC who consumed betel quid and cigarette smoke, SNP rs2274907 was associated with a higher risk of advanced clinical stage (III+IV) and lymph node metastasis. Interestingly, the wild-type TT homozygous genotype was associated with significantly higher *OMNT1* expression levels compared to the AA allele of variant rs2274907. Additionally, *OMNT1* mRNA levels were lower in oral cancer tissues compared to normal tissues, indicating that omentin-1 acts as a negative regulator of oral cancer.

## Introduction

Oral cancer is the fourth most prevalent cancer among Taiwanese men and the ninth most general cancer among men globally 1. The disease is a global health concern since oral squamous cell carcinomas (OSCCs) account for over 90% of all oral cancer malignancies and are linked to high death, recurrence, and early metastasis rates 2. Regular exposure to carcinogens including alcohol, tobacco, and betel nuts is one of the main risk factors for OSCC 3. Regular chewers of betel nuts account for about 86% of Taiwanese patients with oral tumors 4. However, the presence of the human papillomavirus is a risk element for OSCC, and the majority of OSCC linked to HPV is caused by the HPV 16 species 5, 6. Genetic abnormalities brought on by disruptions in DNA repair, carcinogen metabolism, and cell cycle regulation are also linked to OSCC 7.

Adipokines, such as adiponectin, leptin and apelin, have been shown in numerous studies to have regulatory effects on the development of cancer 8. Omentin-1 is a recently documented adipokine with 313 amino acids that is mostly produced in the small intestine and human omental and subcutaneous adipose tissue 9. It has been demonstrated to provide a protective role in reducing proinflammatory cytokines secretion 10. Omentin-1 has been positively correlated with higher levels of anti-inflammatory cytokines, according to earlier experimental research 11, 12. In cancer, serum omentin-1 concentrations are inversely associated with obesity, suggesting that omentin-1 may serve as a marker of tumor progression 13. Furthermore, omentin-1 may function as a tumor-suppressor factor because renal cell carcinoma patients have been found to have reduced serum levels of omentin-1 14.

A difference in a single nucleotide that takes place at a particular location in the genome is known as a single nucleotide polymorphism (SNP) 15. SNP distribution frequency comparisons between patient populations are widely used to forecast the risk and prognosis of diseases, such as cancer 16, 17. There is no information on the relationships between carcinogenic lifestyle variables and omentin-1 (*OMNT1*) gene polymorphisms and OSCC. Therefore, this study examined how a cohort of Taiwanese mans' carcinogenic lifestyle variables and *OMNT1* gene polymorphisms affected their likelihood of developing OSCC. Additionally, we looked at correlations between the *OMNT1* genotypes and the histopathological prognostic variables (metastasis, tumor status, pathological stage, and lymph node metastasis) of OSCC.

## Materials and Methods

### Study participants

This study examined the impact of *OMNT1* variations on the development of OSCC by enrolling 406 male patients with a diagnosis of buccal mucosal cancer and 1192 male controls without cancer who did not have a history of cancer or any oral precancerous symptoms. Carcinogenic lifestyle practices (for instance drinking alcohol, smoking cigarettes, and chewing betel nuts) and demographic information were documented. Those who had smoked at least one cigarette per day for the preceding three months were classified as daily smokers. People who averaged more than two alcoholic beverages each day were classified as alcohol consumers. The 2018 American Joint Committee on Cancer (AJCC) Cancer Staging Manual (8th edition) was used to assess OSCC 18. Tumor cell differentiation was assessed by a pathologist using the AJCC classification criteria. Every procedure used in the study with human subjects met the requirements of the Declaration of Helsinki. Each author read and approved the study after having access to the data. The study inquiry was approved by the Institutional Review Board of Chung Shan Medical University Hospital (IRB No.CS1-21151). Prior to being enrolled in the study, each subject gave written informed consent.

### Selection and genotyping of SNPs

Based on earlier studies in systemic lupus erythematosus, the *OMNT1* SNPs rs2274907, rs35779394, rs4656959, and rs79209815 were chosen 19. The minor allele frequencies for every SNP were more than 5%. QIAamp DNA Blood Kits (Qiagen, CA, USA) were performed to extract genomic DNA from 3 mL peripheral blood samples. The SNPs were subjected to allelic discrimination using previously outlined evaluation methods 20-22. RT-qPCR experiments and RNA isolation were conducted in accordance with our previously published protocols 21, 23, 24.

### Analysis of clinical dataset

To choose oral cancer patients from The Cancer Genome Atlas (TCGA), we performed an extra analysis. In order to identify oral cancer patients whose *OMNT1* gene expression was assessed in each tumor sample, *OMNT1* levels in oral cancer samples obtained from TCGA were examined 17. An extensive public resource for analyzing tissue-specific gene level and modulation is the GTEx portal (gtexportal.org/home/). It supplies quantitative trait loci (QTLs), histological images, and open-access gene expression data 25.

### Statistical analysis

The Fisher's exact test and the Mann-Whitney U test were used to examine the differences between the OSCC and control groups; *p*-values of less than 0.05 were deemed statistically significant. Odds ratios (ORs) and their 95% CIs for correlations between genotype frequencies and OSCC risk were computed performing logistic regression. Age, chewing betel quid, smoking cigarettes, and drinking alcohol were all taken into account while adjusting the ORs. The Statistical Analytic System (SAS) software, version 9.1 for Windows (SAS Institute Inc., CA, USA), was performed to analyze all of the collected data.

## Results

This study examined the relationship between *OMNT1* polymorphisms and the progression of oral carcinogenesis by enrolling 406 Taiwanese male patients with mouth cancer and 1192 cancer-free persons. Clinical and demographic characteristics of both groups were assessed (Table 1). The groups did not differ in age in any noticeable way. Patients with OSCC were more likely to chew betel nuts, smoke cigarettes, and drink alcohol than controls. Nearly all patients were metastasis-free, and 45.1% of patients had clinical stage I/II OSCC and 54.9% had clinical stage III/IV disease according to the AJCC TNM classification and staging system. The proportions of patients classified as T1+T2 or T3+T4 status were similar, and 68.5% had N0 lymph node status and 31.5% had N1+N2+N3 status. 82.8% of patients had OSCC that was moderately or poorly differentiated, while 17.2% had well-differentiated OSCC.

Table 2 displays the genotyping results for the four *OMNT1* SNPs in the patients and controls. After adjusting for cigarette smoking, alcohol intake, and chewing betel quid, none of the genotypes for the four *OMNT1* SNPs in the different groups displayed meaningful correlations (Table 2).

We then looked at how *OMNT1* gene polymorphisms affected the clinicopathologic traits of oral cancer patients. The TA+AA heterozygous genotypes were linked to a markedly greater risk of clinical stage III+IV when compared to the TT genotype at rs2274907 (OR, 1.546; 95% CI, 1.041-2.296; *p*<0.05) (Table 3).

In the subgroup analysis, the TA+AA genotypes of SNP rs2274907 were associated with a higher risk of clinical stage III+IV (OR, 1.666; 95% CI, 1.025-2.707; *p*<0.05) and lymph node metastasis (OR, 1.772; 95% CI, 1.046-3.002; *p*<0.05) in betel quid chewers (Table 4). In addition, the TA+AA heterozygote was linked to a higher risk of clinical stage III+IV (OR 1.667; 95% CI, 1.072-2.591; *p*<0.05) and lymph node metastasis (OR 1.625; 95% CI, 1.004-2.632; *p*<0.05) in smoking OSCC patients with the SNP rs2274907 than the TT wild-type (Table 5).

**Figure 1:**
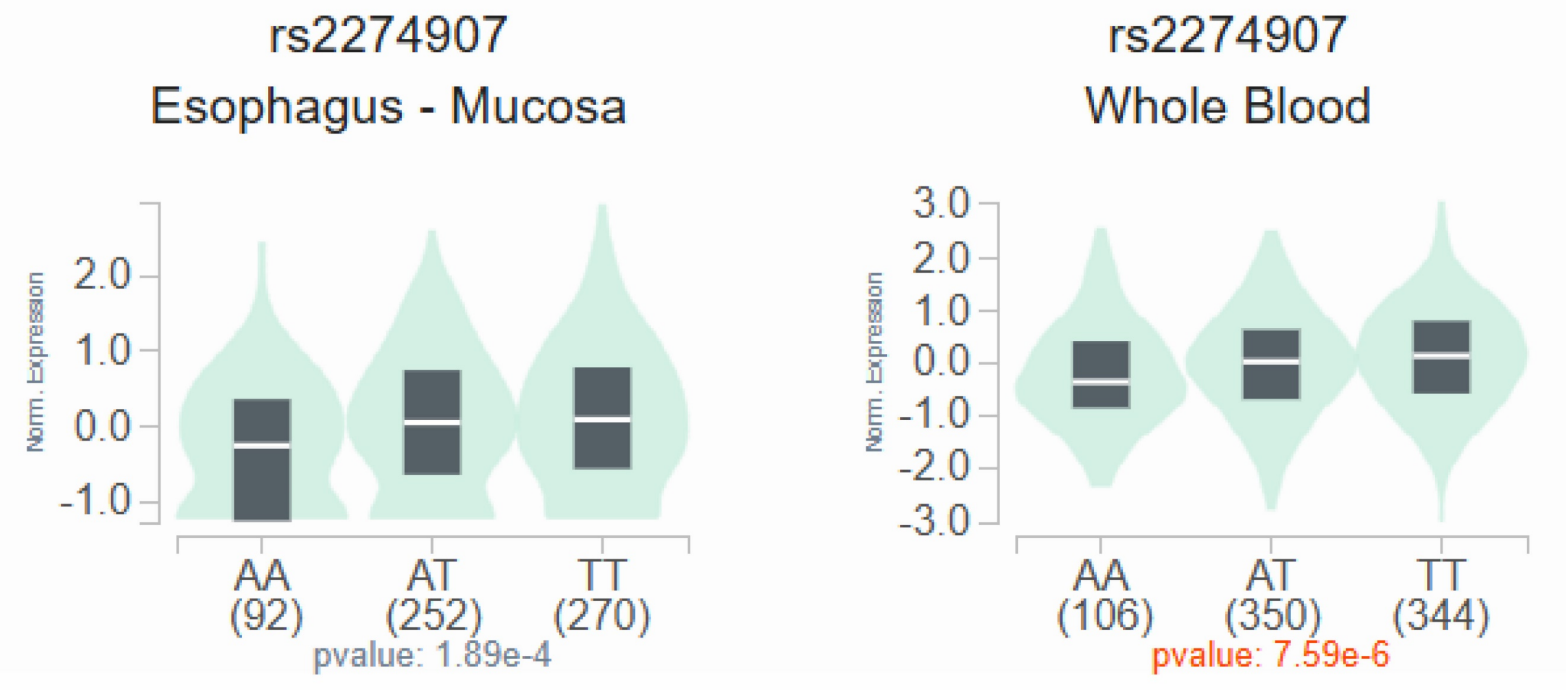
The OMNT1 presents a significant eQTL association with rs2274907 genotypes in mucosa and whole blood from GTEx database.

**Figure 2:**
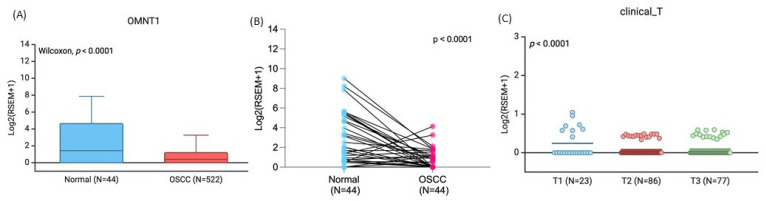
The *OMNT1* mRNA level of oral cancer patients from TCGA database. (A) The omentin-1 levels are lowered in oral cancer patient tissues compared to normal tissues from TCGA database. (B) The paired dot plot indicates that omentin-1 expression was lower in tumor tissues compared to paired normal tissues. (C) Omentin-1 expression levels in oral cancer patients from TCGA database were compared according to the clinical T stage.

According to the GTEx data, patients with the wild-type TT homozygous genotype had considerably higher levels of *OMNT1* than those with the AA allele of variant rs2274907 (*p*<0.05; Fig. 1). Next, we examined *OMNT1* mRNA levels and tumor stage in patients with oral cancer using the TCGA database. It was discovered that tumor tissues have less *OMNT1* expression than normal tissues (Fig. 2A&B). individuals with high-stage tumors (T3) had considerably lower levels of *OMNT1* expression within their tumor tissues than individuals with low-stage tumors (T1) (Fig. 2C).

## Discussion

Over 90% of oral cancers are OSCC 26, and while early examination and remedy of OSCC have greatly improved due to medical advancements, the overall 5-year survival rate is only about 50% 27. The degree of illness at presentation, if the patient has clinically palpable lymph nodes, and histological findings are all significant predictors of outcome 28, 29. Carcinogenic lifestyle choices (for instance drinking alcohol, smoking tobacco, and chewing betel nuts) have an impact on the development of OSCC. Malnutrition reduces quality of life and raises the risk of postoperative complications, infection, and death rate, according to research published in the INSCOC database 30. Therefore, it is important to take into account the relationship between nutritional status and remedy outcomes in OSCC patients. All three of these factors may contribute to the development of OSCC, according to our findings. Recent research on the relationships between gene polymorphisms and the incidence and prognosis of mouth cancer has confirmed this conclusion 31-33. Additionally, carcinogenic lifestyle variables specifically cause epigenetic alterations that raise the incidence of oral tumors 34, 35.

Adipokines, a distinct bioactive peptide secreted by adipose tissues, are involved in many bodily functions 36. In order to ascertain how adipose tissue contributes to the development of inflammation and carcinogens, numerous researchers have been studying this topic for the past 20 years 37. The 34 KD peptide known as omentin-1 has anti-inflammatory and anti-insulin resistance properties 38. Omentin-1 has recently been shown to play a crucial function in cell differentiation and accelerating cancer cell death 39. Numerous related research discovered that the circulation concentrations of omentin-1 in patients with colorectal and renal cell carcinoma varied, indicating that omentin-1 may have a role in the progression of cancer 40. On the other hand, little research has been done on omentin-1 and oral cancer. In order to compare the allelic distributions of four *OMNT1* gene polymorphisms between OSCC patients and healthy, cancer-free participants, we conducted this study. We discovered that patients with the TA+AA genotype of SNP rs2274907 had a higher chance of developing advanced clinical stage (III+IV) than those with the TT genotype. According to our analysis of smokers and betel quid usage, study participants with the TA+AA genotype at rs2274907 had a substantially higher chance of developing advanced clinical stage (III+IV) than those with the wild-type TT allele. Interestingly, GTEx data revealed that the wild-type TT homozygous genotype was associated with significantly higher *OMNT1* expression levels compared to the AA allele of variant rs2274907. The TGGA database confirmed consistent findings, showing that tumor tissues exhibit lower omentin-1 expression than normal tissues, and high-stage tumors have lower omentin-1 expression levels compared to low-stage tumors. Thus, omentin-1 may act as a negative regulator of oral cancer.

"Tumor metastasis" is the term used to describe the process by which the original tumor spreads via the blood or lymphatic system to other tissues or organs 41. It is crucial to comprehend the intricate process of metastasis since blocking lymphangiogenesis may effectively stop tumor growth and metastasis 42-44. Lymphangiogenesis has been associated to tumor growth and metastasis in a number of scholarly publications 44-46. According to our findings, betel quid chewers who have the TA+AA heterozygote and the *OMNT1* SNP rs2274907 are at an increased risk. Similar outcomes were found in smoking patients with oral cancer who had the SNP rs2274907, which was linked to increased lymph node metastases. Omentin-1 function may be influenced by betel quid and cigarette smoke, which may explain why *OMNT1* SNPs are associated with more aggressive effects in betel quid and cigarette smoke consumers but not in non-betel quid, non-smoking OSCC patients. The limitations of the current study should be mentioned, the further investigation is needed, necessitating a larger sample size and longer follow-up period. Moreover, it is essential to validate the current findings in an independent cohort of OSCC cases from Taiwanese populations, as well as other cohorts available in open-access databases.

In conclusion, our investigation reveals associations between *OMNT1* gene variants and advanced clinical stage and lymph node metastasis in OSCC patients. Compared with the TT genotype, the TA+AA genotypes of SNP rs2274907 were associated with an increased risk of progressing advanced clinical stage (III+IV). The wild-type TT homozygous genotype was associated with significantly higher *OMNT1* expression levels compared to the AA allele of variant rs2274907. Additionally, *OMNT1* mRNA levels were lower in oral cancer tissues compared to normal tissues, suggesting that omentin-1 acts as a negative regulator of oral cancer.

## Figures and Tables

**Table 1:** The distributions of demographical characteristics in 1192 controls and 406 male patients with buccal mucosa cancer.

Variable	Controls (N=1192)	Patients (N=406)	p value
Age (yrs)			
< 60	775 (65.0%)	253 (62.3%)	p = 0.326
≥ 60	417 (35.0%)	153 (37.7%)	
Betel quid chewing			
No	995 (83.5%)	134 (33.0%)	
Yes	197 (16.5%)	272 (67.0%)	p < 0.001*
Cigarette smoking			
No	558 (46.8%)	80 (19.7%)	
Yes	634 (53.2%)	326 (80.3%)	p < 0.001*
Alcohol drinking			
No	955 (80.1%)	253 (62.3%)	
Yes	237 (19.9%)	153 (37.7%)	p < 0.001*
Stage			
I+II		183 (45.1%)	
III+IV		223 (54.9%)	
Tumor T status			
T1+T2		209 (51.5%)	
T3+T4		197 (48.5%)	
Lymph node status			
N0		278 (68.5%)	
N1+N2+N3		128 (31.5%)	
Metastasis			
M0		404 (99.5%)	
M1		2 (0.5%)	
Cell differentiation			
Well differentiated		70 (17.2%)	
Moderately or poorly differentiated		336 (82.8%)	

* p value < 0.05 as statistically significant.

**Table 2:** Odds ratio (OR) and 95% confidence interval (CI) of buccal mucosa cancer associated with *OMNT1* genotypic frequencies.

Variable	Controls (N=1192) (%)	Patients (N=406) (%)	AOR (95% CI)	p value
**rs2274907**				
TT	554 (46.5%)	178 (43.8%)	1.000 (reference)	
TA	513 (43.0%)	196 (48.3%)	1.276 (0.974-1.673)	p=0.077
AA	125 (10.5%)	32 (7.9%)	0.789 (0.489-1.272)	p=0.330
TA+AA	638 (53.5%)	228 (56.2%)	1.085 (0.953-1.235)	p=0.220
**rs35779394**				
TT	912 (76.5%)	318 (78.3%)	1.000 (reference)	
TC	255 (21.4%)	86 (21.2%)	1.071 (0.782-1.468)	p=0.669
CC	25 (2.1%)	2 (0.5%)	0.330 (0.071-1.539)	p=0.158
TC+CC	280 (23.5%)	88 (21.7%)	1.007 (0.862-1.175)	p=0.931
**rs4656959**				
AA	566 (47.5%)	175 (43.1%)	1.000 (reference)	
AG	503 (42.2%)	202 (49.8%)	1.295 (0.985-1.865)	p=0.069
GG	123 (10.3%)	29 (7.1%)	0.800 (0.488-1.310)	p=0.375
AG+GG	626 (52.5%)	231 (56.9%)	1.125 (0.988-1.281)	p=0.076
**rs79209815**				
TT	1012 (85.0%)	355 (87.4%)	1.000 (reference)	
TC	168 (14.1%)	50 (12.3%)	0.891 (0.606-1.309)	p=0.556
CC	11 (0.9%)	1 (0.2%)	0.419 (0.048-3.679)	p=0.432
TC+CC	179 (15.0%)	51 (12.6%)	0.932 (0.771-1.126)	p=0.464

The adjusted odds ratio (AOR) with their 95% confidence intervals were estimated by multiple logistic regression models after controlling for betel quid chewing, cigarette smoking, and alcohol drinking.

**Table 3:** Odds ratio (OR) and 95% confidence intervals (CI) of clinical statuses associated with genotypic frequencies of *OMNT1* rs2274907 in 406 male buccal mucosa cancer patients.

Variable	TT (N=178)	TA+AA (N=228)	OR (95% CI)	p value
Clinical Stage				
Stage I+II	91 (51.1%)	92 (40.4%)	1.000 (reference)	p=0.030*
Stage III+IV	87 (48.9%)	136 (59.6%)	1.546 (1.041-2.296)	
Tumor size				
≦ T2	97 (54.5%)	112 (49.1%)	1.000 (reference)	p=0.283
> T2	81 (45.5%)	116 (50.9%)	1.240 (0.837-1.837)	
Lymph node metastasis				
No	130 (73.0%)	148 (64.9%)	1.000 (reference)	p=0.081
Yes	48 (27.0%)	80 (35.1%)	1.464 (0.954-2.247)	
Metastasis				
M0	178 (100.0%)	226 (99.1%)	1.000 (reference)	---
M1	0 (0.0%)	2 (0.9%)	---	
Cell differentiated grade				
Well	27 (15.2%)	43 (18.9%)	1.000 (reference)	p=0.329
Moderate or poor	151 (84.8%)	185 (81.1%)	0.769 (0.454-1.303)	

The odds ratio (OR) with their 95% confidence intervals were estimated by logistic regression models. * p value < 0.05 as statistically significant.

**Table 4:** Clinical statuses and genotypic frequencies of *OMNT1* rs2274907 in 406 buccal mucosa cancer patients who are betel quid chewers or not betel quid chewers.

	Non-Betel Quid Chewers (N=134)				Betel Quid Chewers (N=272)			
Variable	TT (N=60)	TA+AA (N=74)	OR (95% CI)	p value	TT (N=118)	TA+AA (N=154)	OR (95% CI)	p value
**Clinical Stage**								
Stage I+II	31 (51.7%)	33 (44.6%)	1.000 (reference)	0.451	60 (50.8%)	59 (38.3%)	1.000 (reference)	0.039*
Stage III+IV	29 (48.3%)	41 (55.4%)	1.328 (0.671-2.629)		58 (49.2%)	95 (61.3%)	**1.666 (1.025-2.707)**	
**Tumor size**								
≦ T2	34 (56.7%)	40 (54.1%)	1.000 (reference)	0.762	63 (53.4%)	72 (46.8%)	1.000 (reference)	0.278
> T2	26 (43.3%)	34 (45.9%)	1.112 (0.560-2.206)		55 (46.6%)	82 (53.2%)	1.305 (0.807-2.110)	
**Lymph node metastasis**								
No	42 (70.0%)	52 (70.3%)	1.000 (reference)	0.973	88 (74.6%)	96 (62.3%)	1.000 (reference)	0.032*
Yes	18 (30.0%)	22 (29.7%)	0.987 (0.469-2.077)		30 (25.4%)	58 (37.7%)	**1.772 (1.046-3.002)**	
**Cell differentiation**								
Well	4 (6.7%)	9 (12.2%)	1.000 (reference)	0.285	23 (19.5%)	34 (22.1%)	1.000 (reference)	0.603
Moderate or poor	56 (93.3%)	65 (87.8%)	0.516 (0.151-1.766)		95 (80.5%)	120 (77.9%)	0.854 (0.472-1.547)	

The odds ratio (OR) with their 95% confidence intervals were estimated by logistic regression models. * p value < 0.05 as statistically significant.

**Table 5:** Clinical statuses and genotypic frequencies of* OMNT1* rs2274907 in 406 buccal mucosa cancer patients who are smoker and non-smokers.

	Non-smoker (N=80)				Smoker (N=326)			
Variable	TT (N=32)	TA+AA (N=48)	OR (95% CI)	p value	TT (N=146)	TA+AA (N=180)	OR (95% CI)	p value
**Clinical Stage**								
Stage I+II	15 (46.9%)	21 (43.8%)	1.000 (reference)	0.783	76 (52.1%)	71 (39.4%)	1.000 (reference)	0.023*
Stage III+IV	17 (53.1%)	27 (56.3%)	1.134 (0.462-2.786)		70 (47.9%)	109 (60.6%)	**1.667 (1.072-2.591)**	
**Tumor size**								
≦ T2	16 (50.0%)	22 (45.8%)	1.000 (reference)	0.715	81 (55.5%)	90 (50.0%)	1.000 (reference)	0.325
> T2	16 (50.0%)	26 (54.2%)	1.182 (0.482-2.895)		65 (44.5%)	90 (50.0%)	1.246 (0.804-1.931)	
**Lymph node metastasis**								
No	21 (65.6%)	32 (66.7%)	1.000 (reference)	0.923	109 (74.7%)	116 (64.4%)	1.000 (reference)	0.047*
Yes	11 (34.4%)	16 (33.3%)	0.955 (0.371-2.455)		37 (25.3%)	64 (35.6%)	**1.625 (1.004-2.632)**	
**Cell differentiation**								
Well	3 (6.2%)	8 (16.7%)	1.000 (reference)	0.168	25 (17.1%)	35 (19.4%)	1.000 (reference)	0.591
Moderate or poor	30 (93.8%)	40 (83.3%)	0.333 (0.066-1.685)		121 (82.9%)	145 (80.6%)	0.856 (0.485-1.509)	

The odds ratio (OR) with their 95% confidence intervals were estimated by logistic regression models. * p value < 0.05 as statistically significant.

## References

[1] Bray F, Ferlay J, Soerjomataram I, Siegel RL, Torre LA, Jemal A (2018). Global cancer statistics 2018: GLOBOCAN estimates of incidence and mortality worldwide for 36 cancers in 185 countries. CA Cancer J Clin.

[2] Jemal A, Siegel R, Ward E, Hao Y, Xu J, Murray T (2008). Cancer statistics, 2008. CA Cancer J Clin.

[3] Scully C, Field JK, Tanzawa H (2000). Genetic aberrations in oral or head and neck squamous cell carcinoma (SCCHN): 1. Carcinogen metabolism, DNA repair and cell cycle control. Oral Oncol.

[4] Vautrin A, Wesseling M, Wirix-Speetjens R, Gomez-Benito MJ (2021). Time-dependent *in silico* modelling of orthognathic surgery to support the design of biodegradable bone plates. J Mech Behav Biomed Mater.

[5] Gupta K, Metgud R (2013). Evidences suggesting involvement of viruses in oral squamous cell carcinoma. Patholog Res Int.

[6] Scully C, Bagan J (2009). Oral squamous cell carcinoma overview. Oral Oncol.

[7] Bugshan A, Farooq I (2020). Oral squamous cell carcinoma: metastasis, potentially associated malignant disorders, etiology and recent advancements in diagnosis. F1000Research.

[8] Grigoraș A, Amalinei C (2025). The Role of Perirenal Adipose Tissue in Carcinogenesis-From Molecular Mechanism to Therapeutic Perspectives. Cancers.

[9] Schäffler A, Neumeier M, Herfarth H, Fürst A, Schölmerich J, Büchler C (2005). Genomic structure of human omentin, a new adipocytokine expressed in omental adipose tissue. Biochim Biophys Acta.

[10] Rao SS, Hu Y, Xie PL, Cao J, Wang ZX, Liu JH (2018). Omentin-1 prevents inflammation-induced osteoporosis by downregulating the pro-inflammatory cytokines. Bone Res.

[11] Lin YY, Huang CC, Ko CY, Tsai CH, Chang JW, Achudhan D (2025). Omentin-1 modulates interleukin expression and macrophage polarization: Implications for rheumatoid arthritis therapy. Int Immunopharmacol.

[12] Ko CY, Lin YY, Achudhan D, Chang JW, Liu SC, Lai CY (2023). Omentin-1 ameliorates the progress of osteoarthritis by promoting IL-4-dependent anti-inflammatory responses and M2 macrophage polarization. International journal of biological sciences.

[13] Kim JW, Kim JH, Lee YJ (2024). The Role of Adipokines in Tumor Progression and Its Association with Obesity. Biomedicines.

[14] Chinapayan SM, Kuppusamy S, Yap NY, Perumal K, Gobe G, Rajandram R (2022). Potential Value of Visfatin, Omentin-1, Nesfatin-1 and Apelin in Renal Cell Carcinoma (RCC): A Systematic Review and Meta-Analysis. Diagnostics (Basel, Switzerland).

[15] Chanock S (2001). Candidate genes and single nucleotide polymorphisms (SNPs) in the study of human disease. Dis Markers.

[16] Lu HJ, Chuang CY, Su CW, Chen MK, Yang WE, Yeh CM (2022). Role of TNFSF15 variants in oral cancer development and clinicopathologic characteristics. J Cell Mol Med.

[17] Chen KJ, Hsieh MH, Lin YY, Chen MY, Lien MY, Yang SF (2022). Visfatin Polymorphisms, Lifestyle Risk Factors and Risk of Oral Squamous Cell Carcinoma in a Cohort of Taiwanese Males. International journal of medical sciences.

[18] Zanoni DKP, S (2019). G. Shah, J. P. Changes in the 8th Edition of the American Joint Committee on Cancer (AJCC) Staging of Head and Neck Cancer: Rationale and Implications. Curr Oncol Rep.

[19] Zhang TP, Li HM, Li R, Zhang Q, Fan YG, Li XM (2020). Association of omentin-1, adiponectin, and resistin genetic polymorphisms with systemic lupus erythematosus in a Chinese population. Int Immunopharmacol.

[20] Lee HP, Chen PC, Wang SW, Fong YC, Tsai CH, Tsai FJ (2019). Plumbagin suppresses endothelial progenitor cell-related angiogenesis *in vitro* and *in vivo*. Journal of Functional Foods.

[21] Lee HP, Wang SW, Wu YC, Tsai CH, Tsai FJ, Chung JG (2019). Glucocerebroside reduces endothelial progenitor cell-induced angiogenesis. Food and Agricultural Immunology.

[22] Wang B, Hsu CJ, Chou CH, Lee HL, Chiang WL, Su CM (2018). Variations in the AURKA Gene: Biomarkers for the Development and Progression of Hepatocellular Carcinoma. Int J Med Sci.

[23] Lee HP, Wu YC, Chen BC, Liu SC, Li TM, Huang WC (2020). Soya-cerebroside reduces interleukin production in human rheumatoid arthritis synovial fibroblasts by inhibiting the ERK, NF-kappa B and AP-1 signalling pathways. Food and Agricultural Immunology.

[24] Liu SC, Tsai CH, Wu TY, Tsai CH, Tsai FJ, Chung JG (2019). Soya-cerebroside reduces IL-1β-induced MMP-1 production in chondrocytes and inhibits cartilage degradation: implications for the treatment of osteoarthritis. Food and Agricultural Immunology.

[25] Carithers LJ, Moore HM (2015). The Genotype-Tissue Expression (GTEx) Project. Biopreserv Biobank.

[26] Jiang X, Wu J, Wang J, Huang R (2019). Tobacco and oral squamous cell carcinoma: A review of carcinogenic pathways. Tob Induc Dis.

[27] Sim YC, Hwang JH, Ahn KM (2019). Overall and disease-specific survival outcomes following primary surgery for oral squamous cell carcinoma: analysis of consecutive 67 patients. J Korean Assoc Oral Maxillofac Surg.

[28] Montero PH, Patel SG (2015). Cancer of the oral cavity. Surg Oncol Clin N Am.

[29] Alazzawi W, Shahsavari Z, Babaei H, Firouzpour H, Karimi A, Goudarzi A (2022). The evaluation of serum lipid profile and apolipoprotein C-1 in the Iranian patients of Oral Squamous Cell Carcinoma. BioMedicine.

[30] Guo ZQ, Yu JM, Li W, Fu ZM, Lin Y, Shi YY (2020). Survey and analysis of the nutritional status in hospitalized patients with malignant gastric tumors and its influence on the quality of life. Supportive care in cancer: official journal of the Multinational Association of Supportive Care in Cancer.

[31] Shih LC, Tsai CW, Sun KT, Hsu HM, Shen TC, Tsai YT (2019). Association of Caspase-8 Genotypes With Oral Cancer Risk in Taiwan. In vivo.

[32] Shih LC, Li CH, Sun KT, Chen LY, Hsu CL, Hung YW (2018). Association of Matrix Metalloproteinase-7 Genotypes to the Risk of Oral Cancer in Taiwan. Anticancer Res.

[33] Su SC, Hsieh MJ, Liu YF, Chou YE, Lin CW, Yang SF (2016). ADAMTS14 Gene Polymorphism and Environmental Risk in the Development of Oral Cancer. PLoS One.

[34] Wang TH, Hsia SM, Shih YH, Shieh TM (2017). Association of Smoking, Alcohol Use, and Betel Quid Chewing with Epigenetic Aberrations in Cancers. International journal of molecular sciences.

[35] Wu CN, Chang WS, Shih LC, Wang YC, Lee HT, Yu CC (2021). Interaction of DNA Repair Gene XPC With Smoking and Betel Quid Chewing Behaviors of Oral Cancer. Cancer Genomics Proteomics.

[36] Taylor EB (2021). The complex role of adipokines in obesity, inflammation, and autoimmunity. Clinical science (London, England: 1979).

[37] Song YC, Lee SE, Jin Y, Park HW, Chun KH, Lee HW (2020). Classifying the Linkage between Adipose Tissue Inflammation and Tumor Growth through Cancer-Associated Adipocytes. Molecules and cells.

[38] Li Z, Liu B, Zhao D, Wang B, Liu Y, Zhang Y (2017). Omentin-1 prevents cartilage matrix destruction by regulating matrix metalloproteinases. Biomed Pharmacother.

[39] Zhang YY, Zhou LM (2013). Omentin-1, a new adipokine, promotes apoptosis through regulating Sirt1-dependent p53 deacetylation in hepatocellular carcinoma cells. European journal of pharmacology.

[40] Shen XD, Zhang L, Che H, Zhang YY, Yang C, Zhou J (2016). Circulating levels of adipocytokine omentin-1 in patients with renal cell cancer. Cytokine.

[41] Lei PJ, Fraser C, Jones D, Ubellacker JM, Padera TP (2024). Lymphatic system regulation of anti-cancer immunity and metastasis. Front Immunol.

[42] Dieterich LC, Detmar M (2016). Tumor lymphangiogenesis and new drug development. Adv Drug Deliv Rev.

[43] Lin CY, Wang SW, Chen YL, Chou WY, Lin TY, Chen WC (2017). Brain-derived neurotrophic factor promotes VEGF-C-dependent lymphangiogenesis by suppressing miR-624-3p in human chondrosarcoma cells. Cell Death Dis.

[44] Lee HP, Wang SW, Wu YC, Lin LW, Tsai FJ, Yang JS (2020). Soya-cerebroside inhibits VEGF-facilitated angiogenesis in endothelial progenitor cells. Food Agr Immunol.

[45] Paduch R (2016). The role of lymphangiogenesis and angiogenesis in tumor metastasis. Cell Oncol (Dordr).

[46] Su CM, Tang CH, Chi MJ, Lin CY, Fong YC, Liu YC (2018). Resistin facilitates VEGF-C-associated lymphangiogenesis by inhibiting miR-186 in human chondrosarcoma cells. Biochem Pharmacol.

